# Knowledge, Attitude, and Practice of Foot Care and the Risk of Foot Ulcers in Diabetic Patients in Jeddah, Saudi Arabia: A Cross-Sectional Study

**DOI:** 10.7759/cureus.55826

**Published:** 2024-03-08

**Authors:** Hani M Badahdah, Sara F Alshammari, Ohoud M Jassomah, Alhanouf J Alharbi, Dania T Alsiwed, Aseel A Althagafi, Reem A Babakr, Kholoud T Alsiwed, Yousef H Al Zahib, Layan N Alhelali, Shoog H Alzahib

**Affiliations:** 1 Podiatric and Foot and Ankle Surgery, Dr. Khalid Edrees Medical Center, Jeddah, SAU; 2 Medicine, Fakeeh College for Medical Sciences, Jeddah, SAU; 3 Medicine, Arabian Gulf University, Manama, BHR; 4 Medicine, King Abdulaziz University, Jeddah, SAU; 5 Family Medicine, Ministry of Health, Jeddah, SAU; 6 Medicine, Ibn Sina National College for Medical Science, Jeddah, SAU

**Keywords:** jeddah, diabetes, foot care, practice, attitude, knowledge

## Abstract

Background and objective: Diabetic patients in Saudi Arabia are often underinformed about proper diabetic foot care. This study aims to determine the knowledge, attitude, and practice of the risk factors of diabetic foot ulcers among diabetic patients in the Jeddah region of Saudi Arabia.

Materials and methods: A cross-sectional study was done on 210 diabetic patients attending the international diabetic center in Jeddah, Saudi Arabia, during the study period. A validated questionnaire was used to collect data about participants' demographics and knowledge, attitudes, and practices of diabetic foot care.

Results: Of the participants, 77 (36.7%) were aged 51-60 years; 161 (76.7%) were male; 153 (72.9%) had a university education; and approximately 138 (65%) had type 2 DM. A majority, 190 (90.5%), acknowledged that DM patients might have reduced foot sensation; 204 (97.1%) agreed that diabetics could develop gangrene; 188 (89.5%) concurred that poor foot sensation increases the risk of foot ulcers; and 193 (91.9%) agreed that poor blood flow to the feet heightens this risk. Among them, 152 (72.4%) demonstrated good knowledge about foot care and the risk of foot ulcers; eight (3.8%) exhibited a positive attitude; and 95 (45.2%) showed a good level of practice. Good knowledge was significantly higher among married patients, and good practice was notably higher among older patients (>50 years). A significant positive correlation was found between knowledge scores and both attitude and practice scores.

Conclusion: The study revealed adequate knowledge and practice of foot care and the risk of foot ulcers among diabetic patients. However, a high percentage of negative attitudes toward these issues were observed.

## Introduction

Diabetes mellitus (DM), the most prevalent health issue worldwide, is a chronic, progressive metabolic disease characterized by hyperglycemia due to insulin deficiency or resistance [1,2]. Diabetic foot, a serious complication of diabetes, results from peripheral artery disease affecting diabetic patients' feet [3,4]. Diabetic foot disease is marked by recurrent infections, foot ulcers, and neuropathic osteoarthropathy [5]. These long-term complications significantly impact the quality of life of diabetics [6-8].

A study found that diabetic foot complications were present among 3.3% of patients, with foot ulcers at 2.05%, gangrene at 0.19%, and amputations at 1.06% [9]. Another study found that diabetic foot grade ≥4 (4 or 5) according to Wagner's classification was present in 24% of amputee patients [10]. Despite the lack of worldwide epidemiological data on diabetic foot ulceration, a meta-analysis involving over 800,000 participants from 67 studies worldwide revealed a global prevalence of diabetic foot ulceration. The prevalence was 6.3%, with the highest in North America (13%) and the lowest in Africa (3%). Asia had a prevalence of 5.5%, Europe 5.1%, and Oceania 3.0%. Belgium had the highest national prevalence (16.6%), followed by Canada (14.8%) and the United States (13%), while Australia had the lowest prevalence (1.5%) [11].

The World Health Organization (WHO) reports Saudi Arabia as having the second-highest diabetes prevalence in the Middle East and the seventh-highest globally [12]. In this region, the prevalence rate of diabetic foot ulceration was nearly 2.3%, as per the aforementioned meta-analysis [11]. Diabetic foot diseases arise from various factors, including reduced joint mobility, foot abnormalities, pressure or trauma to the feet, and peripheral vascular or neuropathic diseases [13]. A study in Pakistan showed that most participants received foot care information from doctors, media, and colleagues, with doctors being the primary source (61.1%), followed by the media (20%) and colleagues (2.2%) [14].

A previous study evaluated diabetic foot care knowledge, attitudes, previous education, and practices [15]. Nearly half of the participants (55.1%) had good knowledge of diabetic foot care and risks, yet attitudes scored lower than knowledge. There was a notable disparity in knowledge, practice, and education about DM foot care. Almost 69% of participants had no prior education, and 56.5% scored 6-10 out of 15 in the diabetic foot practice assessment. Females outperformed males in knowledge, attitude, and practice [15].

In parallel, a 2020 study in Alkharj, Saudi Arabia, found that although the scores indicated good knowledge and attitudes among diabetic patients, there was a notable lack of practice in DM foot management [2]. A similar study done in 2021 in the Aseer region found good knowledge of diabetic foot care among 67.4% of patients [16].

Despite some studies indicating a high level of knowledge among diabetic patients, there still seems to be a lack of sufficient awareness regarding proper DM foot care. Consequently, increasing awareness and emphasizing correct diabetic foot management practices are essential to preventing complications such as foot ulcers and amputations.

This study aims to assess knowledge, attitudes, and practices of the risk factors for diabetic foot ulcers among diabetic patients in the Jeddah region of Saudi Arabia.

## Materials and methods

Study design, location, and time

This was a cross-sectional study done at the international diabetic center in Jeddah City, Saudi Arabia, from June to August 2023.

Study participants

The inclusion criteria were Saudi and non-Saudi diabetic patients of both genders with an age of ≥20 years who attended the diabetic center during the period of the study. The exclusion criteria were patients below the age of 20 years, those already suffering from diabetic foot, amputated foot, or foot ulcers, or those who refused to share in the study.

Sample size

A total of 283 diabetic patients attended the center during the study period. After the application of the inclusion criteria, 201 patients were eligible to participate in the study, with a response rate of 74.2% [17].

Data collection

A validated questionnaire that was used in a previous study [18] was completed by the study participants through personal interviews. The questionnaire included four parts. The first one consisted of sociodemographic information, and the second, third, and fourth sections included items to assess knowledge, attitude, and practice of diabetic foot care, respectively. For the knowledge questions, a score of "1" was given for a correct answer and "0" for an incorrect answer. Participants were considered to have poor knowledge if they answered less than 50% correctly, fair knowledge for 50% to 75% correct answers, and good knowledge for more than 75% correct answers [19]. The same scoring was applied to categorize negative, fair, and positive attitudes and poor, fair, and good practices [19].

Ethical considerations

Ethical approval for the study was obtained from the Institutional Review Board of the Fakeeh College for Medical Sciences in Jeddah, Saudi Arabia (approval number: 380/IRB/2022). All participants were informed about the objectives of the study and received a proper explanation of the items in the questionnaire.

Data analysis

Data were analyzed using SPSS Statistics version 26 (IBM Corp. Released 2019. IBM SPSS Statistics for Windows, Version 26.0. Armonk, NY: IBM Corp.). The association between variables was assessed using the Chi-squared test (χ2) for qualitative data, expressed as numbers and percentages. Quantitative data were expressed as mean and standard deviation (mean ± SD). Correlation analysis was performed using Spearman's test, with a p-value of <0.05 considered statistically significant.

## Results

Table 1 shows that among the participants, 77 (36.7%) were aged between 51 and 60 years, 161 (76.7%) were male, and 83.8% were married. Of these, 101 (48.1%) were retired, and 153 (72.9%) had a university-level education.

**Table 1:** Distribution of studied participants according to their demographic characteristics (N=210) N.B.: Data has been represented as numbers and percentages

Variable	N (%)
Age (years)	
20–30	13 (6.2)
31–40	18 (8.6)
41–50	45 (21.4)
51–60	77 (36.7)
>60	57 (27.1)
Gender	
Female	49 (23.3)
Male	161 (76.7)
Marital status	
Divorced	5 (2.4)
Married	176 (83.8)
Single	23 (11)
Widower	6 (2.9)
Occupation	
Government sector	60 (28.6)
Housewife	14 (6.7)
Private sector	24 (11.4)
Retired	101 (48.1)
Other	11 (5.2)
Education level	
Reads and writes	5 (2.4)
Primary school	2 (1)
Preparatory school	14 (6.7)
Secondary school	36 (17.1)
University	153 (72.9)

The majority of participants had type 2 DM (65.7%), with 85 (40.5%) having a disease duration of less than 10 years and 31% having a DM duration of 10-29 years. Additionally, 151 (71.9%) visited a diabetes clinic 1-4 times per year on average, 13.8% visited it 5-6 times, 6.7% visited it 7-10 times, and 7.6% visited it ≥11 times. The participants' responses to the items in the knowledge, attitude, and practice survey are illustrated in Table 2. Regarding their knowledge, it was found that most participants, 164 (78.1%), agreed that DM might cause poor blood flow in the feet, and 90.5% agreed that DM patients might experience weak sensations in their feet. Additionally, 204 (97.1%) agreed that diabetics might develop gangrene, 188 (89.5%) agreed that poor sensation in the feet increases susceptibility to foot ulcers, and 193 (91.9%) agreed that poor blood flow to the feet also increases this risk. However, only 34 (16.2%) agreed that diabetics might develop ulcers on their feet. Regarding attitudes, 62 (29.5%) of the patients had attended an awareness class on foot care. Among them, 113 (53.8%) and 66 (31.4%) were taught by doctors and nursing staff, respectively. About 45% (N=95, 45.2%) reported that diabetics should self-examine their feet, and the majority (N=205, 97.6%) reported that diabetics could enjoy a normal lifespan by regulating blood sugar levels. Nearly all participants (N=208, 99%) recognized the importance of nutrition in controlling blood sugar levels. Only 28 (13.3%) had lesions or scratches on their feet, of whom 20 (71.6%) stated they would visit a healthcare center if they had wounds on their feet. In terms of practice, 93 (44.3%) checked their feet daily for red spots, swelling, or wounds, while 199 (94.8%) reported daily washing of their feet. More than half (N=199, 56.7%) dried their feet and between their toes after washing, and 59.5% used moisturizers. Most (N=176, 83.8%) avoided walking barefoot; 164 (78.1%) checked their shoes before wearing them; about 78% (N=164, 78.1%) tested their water temperature before showering or washing their feet; and 81.9% trimmed their toenails straight and filed the edges.

**Table 2:** Participants' responses to individual items of the knowledge, attitude, and practice survey N.B.: Data has been represented as numbers and percentages

Variable	No (N (%))	Yes (N (%))
Knowledge of participants in foot care		
Diabetics may have poor blood flow in the feet	46 (21.9)	164 (78.1)
Diabetic patients may have weak sensation in the feet	20 (9.5)	190 (90.5)
Diabetics may get ulcers on the feet	176 (83.8)	34 (16.2)
Diabetics may develop gangrene	6 (2.9)	204 (97.1)
With poor sensation in the feet, one may be more prone to foot ulcers	22 (10.5)	188 (89.5)
With poor blood flow to the feet, one may be more prone to foot ulcers	17 (8.1)	193 (91.9)
Attitude of participants toward foot care		
Attended a class on foot care awareness	148 (70.5)	62 (29.5)
Learned foot care from a doctor	97 (46.2)	113 (53.8)
Learned foot care from the nursing staff	144 (68.6)	66 (31.4)
Believe diabetics should examine their feet independently	115 (54.8)	95 (45.2)
Believe controlling blood sugar levels can extend life expectancy	5 (2.4)	205 (97.6)
Recognize nutrition as important for controlling blood sugar levels	2 (1)	208 (99)
Had lesions or scratches on feet	182 (86.7)	28 (13.3)
If yes, actions taken for wounds on feet (N=28)		
Visited a healthcare center		20 (71.6)
Waited for a hospital appointment		4 (14.2)
Used herbal medicine		3 (10.7)
Regretted going to the hospital		1 (3.5)
The practice of participants regarding foot care		
Check feet daily for red spots, swelling, or wounds	117 (55.7)	93 (44.3)
Wash feet daily	11 (5.2)	199 (94.8)
Dry feet and between toes after washing	91 (43.3)	119 (56.7)
Use moisturizers for feet	85 (30.5)	125 (59.5)
Avoid walking barefoot	34 (16.2)	176 (83.8)
Check shoes before wearing	46 (21.9)	164 (78.1)
Test water temperature before showering or washing feet	46 (21.9)	164 (78.1)
Trim toenails straight and file edges	38 (18.1)	172 (81.9)

The mean knowledge score was 4.63 ± 1.05, while the mean scores for attitude and practice were 3.79 ± 1.57 and 5.77 ± 1.94, respectively. The majority of the participants (152 or 72.4%) possessed good knowledge about foot care and the risk of foot ulcers. Meanwhile, 45 participants (21.4%) had a fair level of knowledge, and 13 participants (6.2%) had a poor level of knowledge. Regarding the participants' attitudes, only eight (3.8%) exhibited a positive attitude, while 102 (48.6%) had a fair attitude, and 100 (47.6%) displayed a negative attitude. In terms of practice level, 95 participants (45.2%) demonstrated good practice, 86 (41%) had fair practice, and 29 (13.8%) showed poor practice.

Table 3 reveals that a good knowledge level was significantly more prevalent among married patients (p≤0.05). Conversely, there was no significant relationship between participants' level of knowledge and other demographics, type of DM, duration of DM, or frequency of yearly clinic visits (p≥0.05).

**Table 3:** Relationship between participants' knowledge level of foot care, risk of foot ulcer, and their demographic characteristics (N=210) N.B.: The p-value is considered significant when <0.05

Variable	Knowledge level			χ2	p-value
	Poor N (%)	Fair N (%)	Good N (%)		
Age (years)					
20–30	1 (7.7)	2 (4.4)	10 (6.6)	3.96	0.113
31–40	2 (15.4)	5 (11.1)	11 (7.2)		
41–50	3 (23.1)	14 (31.1)	28 (18.4)		
51–60	6 (46.2)	8 (17.8)	63 (41.4)		
>60	1 (7.7)	16 (35.6)	40 (26.3)		
Gender					
Female	3 (23.1)	11 (24.4)	35 (23)	0.04	0.98
Male	10 (76.9)	34 (75.6)	117 (77)		
Marital status					
Divorced	1 (7.7)	1 (2.2)	3 (2)	13.52	0.035
Married	7 (53.8)	38 (84.4)	131 (86.2)		
Single	5 (38.5)	4 (8.9)	14 (9.2)		
Widower	0 (0.0)	2 (4.4)	4 (2.6)		
Occupation					
Government sector	3 (23.1)	13 (28.9)	44 (28.9)	5.3	0.727
Housewife	1 (7.7)	4 (8.9)	9 (5.9)		
Private sector	2 (15.4)	2 (4.4)	7 (4.6)		
Retired	2 (15.4)	7 (15.6)	15 (9.9)		
Other	5 (38.5)	19 (42.2)	77 (50.7)		
Education level					
Reads and writes	0 (0.0)	2 (4.4)	3 (2)	9.89	0.272
Primary school	1 (7.7)	0 (0.0)	1 (0.7)		
Preparatory school	0 (0.0)	4 (8.9)	10 (6.6)		
Secondary school	3 (23.1)	6 (13.3)	27 (17.8)		
University	9 (69.2)	33 (73.3)	111 (73)		
Diabetes type					
Type 1	5 (38.5)	18 (40)	49 (32.2)	1.03	0.596
Type 2	8 (61.5)	27 (60)	103 (67.8)		
Diabetes mellitus (years)					
<10	10 (76.9)	17 (37.8)	58 (38.2)	7.79	0.1
10–29	2 (15.4)	14 (31.1)	45 (29.6)		
≥20	1 (7.7)	14 (31.1)	49 (32.2)		
Average number of diabetes clinic visits per year					
1–4 times	9 (69.2)	33 (73.3)	109 (71.7)	7.23	0.3
5–6 times	0 (0.0)	6 (13.3)	23 (15.1)		
7–10 times	2 (15.4)	1 (2.2)	11 (7.2)		
≥11 times	2 (15.4)	5 (11.1)	9 (5.9)		

Table 4 indicates that there was no significant relationship between participants' attitude levels and their demographics, type of DM, duration of DM, or frequency of yearly clinic visits (p≥0.05).

**Table 4:** Relationship between participants' attitude level of foot care, risk of foot ulcers, and their demographic characteristics (N=210) N.B.: The p-value is considered significant when <0.05

Variable	Attitude level			χ2	p-value
	Negative N (%)	Fair N (%)	Positive N (%)		
Age (years)					
20–30	4 (4)	7 (6.9)	2 (25)	7.91	0.442
31–40	9 (9)	8 (7.8)	1 (12.5)		
41–50	20 (20)	23 (22.5)	2 (25)		
51–60	41 (41)	35 (34.3)	1 (12.5)		
>60	26 (26)	29 (28.4)	2 (25)		
Gender					
Female	26 (26)	21 (20.6)	2 (25)	0.84	0.657
Male	74 (74)	81 (79.4)	6 (75)		
Marital status					
Divorced	2 (2)	3 (2.9)	0 (0.0)	2.96	0.814
Married	86 (86)	84 (82.4)	6 (75)		
Single	10 (10)	11 (10.8)	2 (25)		
Widower	2 (2)	4 (3.9)	0 (0.0)		
Occupation					
Government sector	29 (29)	27 (26.5)	4 (50)	6.87	0.55
Housewife	9 (9)	5 (4.9)	0 (0.0)		
Private sector	6 (6)	5 (4.9)	0 (0.0)		
Retired	9 (9)	13 (12.7)	2 (25)		
Other	47 (47)	52 (51)	2 (25)		
Education level					
Reads and writes	3 (3)	2 (2)	0 (0.0)	3.34	0.911
Primary school	1 (1)	1 (1)	0 (0.0)		
Preparatory school	6 (6)	7 (6.9)	1 (12.5)		
Secondary school	20 (20)	13 (15.7)	0 (0.0)		
University	70 (70)	76 (74.5)	7 (87.5)		
Diabetes type					
Type 1	33 (33)	36 (35.3)	3 (37.5)	0.15	0.925
Type 2	67 (67)	66 (64.7)	5 (62.5)		
Diabetes mellitus (years)					
<10	41 (41)	40 (39.2)	4 (50)	1.82	0.769
10–29	34 (34)	29 (298.2)	2 (25)		
≥20	25 (25)	33 (32.4)	2 (25)		
Average number of diabetes clinic visits per year					
1–4 times	77 (77)	68 (66.7)	6 (75)	5.83	0.442
5–6 times	12 (12)	16 (15.7)	1 (12.5)		
7–10 times	3 (3)	10 (9.8)	1 (12.5)		
≥11 times	8 (8)	8 (7.8)	0 (0.0)		

It was observed that a good practice level was significantly higher among patients aged over 50 years (p≤0.05). However, there was no significant relationship between participants' practice levels and their demographics (other than age), type of DM, duration of DM, or frequency of yearly clinic visits (p≥0.05) (Table 5).

**Table 5:** Relationship between participants' practice level of foot care, risk of foot ulcers, and their demographic characteristics (N=210) N.B.: The p-value is considered significant when <0.05

Variable	Practice level			χ2	p-value
	Poor N (%)	Fair N (%)	Good N (%)		
Age (years)					
20–30	3 (10.3)	4 (4.7)	6 (6.3)	15.82	0.045
31–40	3 (10.3)	3 (3.5)	12 (12.6)		
41–50	7 (24.1)	25 (29.1)	13 (13.7)		
51–60	8 (27.6)	37 (43)	32 (33.7)		
>60	8 (27.6)	17 (19.8)	32 (33.7)		
Gender					
Female	4 (13.8)	22 (25.6)	23 (24.2)	1.75	0.415
Male	25 (86.2)	64 (74.4)	72 (75.8)		
Marital status					
Divorced	0 (0.0)	0 (0.0)	5 (5.3)	9.99	0.125
Married	22 (75.9)	77 (89.5)	77 (81.1)		
Single	6 (20.7)	7 (8.1)	10 (10.5)		
Widower	1 (3.4)	2 (2.3)	3 (3.2)		
Occupation					
Government sector	7 (24.1)	32 (37.2)	21 (22.1)	13.67	0.091
Housewife	3 (10.3)	4 (4.7)	7 (7.4)		
Private sector	4 (13.8)	4 (4.7)	3 (3.2)		
Retired	5 (17.2)	8 (9.3)	11 (11.6)		
Other	10 (34.5)	38 (44.2)	53 (55.8)		
Education level					
Reads and writes	2 (6.9)	1 (1.2)	2 (2.1)	7.75	0.457
Primary school	0 (0.0)	1 (1.2)	1 (1.1)		
Preparatory school	2 (6.9)	7 (8.1)	5 (5.3)		
Secondary school	8 (27.6)	11 (12.8)	17 (17.9)		
University	17 (58.6)	66 (76.6)	70 (73.7)		
Diabetes type					
Type 1	8 (27.6)	29 (33.7)	35 (36.8)	0.86	0.649
Type 2	21 (72.4)	57 (66.3)	60 (63.2)		
Diabetes mellitus (years)					
<10	11 (37.9)	37 (43)	37 (38.9)	3.13	0.535
10–29	8 (27.6)	30 (34.9)	27 (28.4)		
≥20	10 (34.5)	19 (22.1)	31 (32.6)		
Average number of diabetes clinic visits per year					
1–4 times	24 (82.8)	65 (75.6)	62 (65.3)	5.41	0.492
5–6 times	2 (6.9)	9 (10.5)	18 (18.9)		
7–10 times	1 (3.4)	6 (7)	7 (7.4)		
≥11 times	2 (6.9)	6 (7)	8 (8.4)		

Table 6 shows a significant positive correlation was found between knowledge scores and both attitude scores (r=0.3, p<0.001) and practice scores (r=0.14, p=0.039). Furthermore, there was a significant positive correlation between attitude scores and practice scores (r=0.36, p≤0.00).

**Table 6:** A significant positive correlation was found between knowledge scores and both attitude scores (r=0.3, p<0.001) and practice scores (r=0.14, p=0.039). Furthermore, there was a significant positive correlation between attitude scores and practice scores (r=0.36, p≤0.00) N.B.: The p-value is considered significant when <0.05

Variable	Knowledge scores	
	r	p-value
Attitude scores	0.3	<0.001
Practice scores	0.14	0.039
Variable	Attitude scores	
	r	p-value
Practice scores	0.36	<0.001

## Discussion

The current study aimed to assess the knowledge, attitudes, and practices of diabetic patients of the risk factors for diabetic foot ulcers in the Jeddah region of Saudi Arabia. The findings indicate that the majority of participants (72.4%) had a high level of knowledge about foot care and the risk of foot ulcers. This level surpasses that found in a 2019 study in Jeddah, where only 38% of patients demonstrated good knowledge of diabetic foot care [20]. This increased awareness could be attributed to the high educational level among the studied participants, where 72.9% of our participants had a university level of education compared to 23.4% of the Jeddah study.

A high level of knowledge was also reported among diabetic patients studied in the Asir region. Their responses revealed that 77.5% knew diabetics could develop gangrene in the foot, 74.9% were aware of the risk of foot ulcers, 66.7% understood that diabetes could reduce blood flow to the feet, and 66.6% recognized that diabetic patients might suffer from a lack of sensation in the feet [16].

Nearly one-third of the patients in our study (29.5%) attended a foot-care awareness class. Doctors taught 53.8% of these patients, while nursing staff educated the remaining 31.4% on foot care. Wazqar et al. found more encouraging results, with 49.1% of diabetic patients receiving information through diabetic foot care education classes [21]. In contrast, an Italian study reported that 28% of participants had not received any foot education [22]. These differences could be attributed to differences in study populations, settings, designs, tools used to measure knowledge, attitude, and practice, and data collection methods. Furthermore, differences in the performance of healthcare systems across countries could explain the disparities.

Our study found that 44.3% of participants regularly checked their feet for new red spots, swelling, or wounds, and 94.8% washed their feet daily. Over half (56.7%) dried their feet and between their toes after washing, and 59.5% moisturized them. Furthermore, 78.1% checked the water temperature before showering or washing their feet, and 81.9% trimmed their toenails straight and filed the edges.

A previous Saudi study conducted in Makkah found that the majority of participants (76.9%) inspected their feet, and 37.7% examined their feet daily. Almost all participants (96.3%) regularly washed their feet [21]. Another study in Saudi Arabia, the Asir region, noted a high level of practice among diabetic patients: 82.2% of the patients washed their feet daily, 29.5% regularly wore cotton socks, and 39.6% occasionally wore stockings. Only 19.2% regularly walked barefoot, with 41.7% doing so occasionally [16].

International research, including a study in Chandigarh, indicates that 63.3% of diabetic patients regularly wash their feet [23]. This disparity might be attributed to Islamic rituals, as the participants in the current study religiously performed foot washing during ablution before praying, a practice beneficial for foot care. However, many other studies from India have shown poor foot care practices [24].

Meanwhile, a significant majority of study participants (83.8%) did not walk barefoot upon waking, and 78.1% checked their shoes before wearing them. A previous study in Makkah reported that 64.3% of respondents always checked their shoes before wearing them [21]. In contrast, a 2019 study in Jeddah found that only 22% adhered to good diabetic foot care practices [20]. This study’s findings are consistent with those by Hasnain et al. [25], where 73.3% of participants checked their shoes before wearing them.

Patients with poor diabetic foot care practices are more likely to develop complications like foot ulcers [26]. Good foot care practices, on the other hand, not only reduce common issues such as corns and callosities but also accelerate the healing of foot ulcers [27]. Nearly 45% of the participants in this study had a high level of practice. This could be linked to the participants performing religious rituals, which include activities beneficial for foot care, such as washing feet before prayers, often without realizing their health benefits [21].

Foot care education is crucial in preventing lower leg amputations, with physicians playing a key role in enhancing foot care knowledge and practices. Consequently, there is a strong correlation between patients’ knowledge, practices, and attitudes of their physicians [22,26-29]. In this study, married patients showed significantly higher levels of knowledge, while older patients, those over 50 years, had significantly higher practice levels. The higher compliance rate among older patients could be due to their concerns about diabetes-related complications impacting their future lives [16].

A previous study conducted in Jeddah found no association between foot care knowledge and practice and educational level or socioeconomic status [20]. The Desalu et al. study also identified a non-significant association between knowledge level and patient demographics, except for marital status. In their study, females exhibited better foot care knowledge and practice than males. This finding aligns with our results, indicating a non-significant relationship between foot care knowledge and practice and educational and socioeconomic levels [30].

Our study revealed a significant positive correlation between knowledge scores and both attitude and practice scores, a connection similarly noted in a previous Saudi study [21]. Considering the critical role of health behaviors in diabetes, such as self-management and health-related lifestyle choices [31,32], the link between educational attainment and health behaviors becomes particularly relevant. Karter et al. [33] examined these associations in diabetes patients under managed care, focusing on behaviors such as smoking, physical activity, blood glucose self-monitoring, foot self-examination, and health-seeking behaviors. Shah et al. [34] also highlighted the increasing evidence supporting patient education as the most effective method for reducing diabetes complications and managing the disease.

The American Diabetes Association recommends that diabetic patients undergo annual evaluations of their knowledge, skills, and behaviors related to diabetes management [35].

Limitations 

The use of a cross-sectional study design could reveal the associations between variables without observing casual relationships. The unequal distribution of both genders in the present study was another limitation.

## Conclusions

The study’s findings indicate that the majority of participants possess a high level of knowledge regarding foot care and the risks of foot ulcers. However, their attitudes and practices of foot care were generally negative, despite a significant positive correlation between knowledge and both attitude and practice scores. The attitudes and practices of diabetics toward foot care can be improved. Awareness programs in the Kingdom of Saudi Arabia for the early detection and treatment of diabetic foot issues could facilitate this improvement. Additionally, regular evaluations of patients’ performances are essential to promoting healthy practices.

## Appendices

**Table 7:** Sociodemographic characteristics of the participants DM: diabetes mellitus, Hx: history

	Parameter
Gender	Male, female
Age	Less than 20, 20-30 years old, 31-40 years old, 41-50 years old, 51-60 years old , more than 60
Social status	Single, married, divorced, widowed
Occupation	Government sector, private sector, retired, housewife, other
Education level	Illiterate, read and write, primary, preparatory, secondary, university
Duration of disease with DM	Less than 10 years, from 11 to 19, 20 years and over
Which DM do you have	Type 1, type 2
Do you have any hx of DM foot complications	Toe amputation, metatarsal amputation, amputation below knee, amputation above knee, heel ulcers, foot ulcers, don’t have
Average number of diabetes clinic visits per year	1-4 times, 5 to 6 times, 7 to 10 times, 11 times and more

**Table 8:** Knowledge of participants in foot care

Parameter	Yes	No
Diabetics may have poor blood flow in the feet		
Diabetic patients may have weak sensation in the feet		
Diabetics may get ulcers on the feet		
Diabetics may get gangrene		
With poor feeling in the feet, you may be more prone to foot ulcers		
With poor blood flow to your feet, you may be more prone to foot ulcers		

**Table 9:** Attitude of participants in foot care

Parameter	Yes	No
Attended an awareness class on how to take care of their feet		
Taught how to take care of their feet from a doctor		
Taught how to take care of their feet from the nursing staff		
Diabetics should examine their feet by themselves		
Diabetics can enjoy an ordinary lifetime by regulating blood sugar levels		
Nutrition is an important factor in controlling blood sugar levels		
Had lesions and scratches on their feet, and what they do if they had wounds and wounds on their feet		
Goes to the healthcare center		
Goes to the emergency		
Waits for hospital appointment		
Uses herbal medicine		
Went to the hospital and wished they didn't go		

**Table 10:** Practice of participants in foot care

Parameter	Yes	No
Check their feet daily and look for any new red spots/swelling/wounds		
Wash their feet daily		
Dry their feet and between the toes after washing them		
Use moisturizers to moisturize the feet		
Does not walk barefoot		
Check shoes before wearing them		
Check water temperature before taking a shower and washing their feet		
Trim toenails straight and file edges		
